# Synergistic roles of Wnt modulators *R-spondin2* and *R-spondin3* in craniofacial morphogenesis and dental development

**DOI:** 10.1038/s41598-021-85415-y

**Published:** 2021-03-12

**Authors:** Nora Alhazmi, Shannon H. Carroll, Kenta Kawasaki, Katherine C. Woronowicz, Shawn A. Hallett, Claudio Macias Trevino, Edward B. Li, Roland Baron, Francesca Gori, Pamela C. Yelick, Matthew P. Harris, Eric C. Liao

**Affiliations:** 1 Harvard School of Dental Medicine, Boston, MA USA; 2 Center for Regenerative Medicine, Massachusetts General Hospital, Boston, MA USA; 3 Shriners Hospital for Children, Boston, MA USA; 4 Department of Medicine, Harvard Medical School, Boston, MA USA; 5 Department of Genetics, Harvard Medical School, Boston, MA USA; 6 Department of Orthopedics, Boston Children’s Hospital, Boston, MA USA; 7 Department of Orthodontics, Division of Craniofacial and Molecular Genetics, Tufts University School of Dental Medicine, Boston, MA USA; 8 Division of Plastic and Reconstructive Surgery, Massachusetts General Hospital, Boston, MA USA

**Keywords:** Model vertebrates, Gene expression analysis

## Abstract

Wnt signaling plays a critical role in craniofacial patterning, as well as tooth and bone development. *Rspo2* and *Rspo3* are key regulators of Wnt signaling. However, their coordinated function and relative requirement in craniofacial development and odontogensis are poorly understood. We showed that in zebrafish *rspo2* and *rspo3* are both expressed in osteoprogenitors in the embryonic craniofacial skeleton. This is in contrast to mouse development, where *Rspo3* is expressed in osteoprogenitors while *Rspo2* expression is not observed. In zebrafish, *rspo2* and *rspo3* are broadly expressed in the pulp, odontoblasts and epithelial crypts. However, in the developing molars of the mouse, *Rspo3* is largely expressed in the dental follicle and alveolar mesenchyme while *Rspo2* expression is restricted to the tooth germ. While *Rspo3* ablation in the mouse is embryonic lethal, zebrafish *rspo3*-/- mutants are viable with modest decrease in Meckel’s cartilage rostral length. However, compound disruption of *rspo3* and *rspo2* revealed synergistic roles of these genes in cartilage morphogenesis, fin development, and pharyngeal tooth development. Adult *rspo3*^−/−^ zebrafish mutants exhibit a dysmorphic cranial skeleton and decreased average tooth number. This study highlights the differential functions of *Rspo2* and *Rspo3* in dentocranial morphogenesis in zebrafish and in mouse.

## Introduction

The Wnt signaling pathway plays a major role in skeletal patterning and differentiation during embryonic development, and in maintaining postnatal bone homeostasis^1–3^. Impairment and potentiation of Wnt signaling affects overall bone mass and density^1–3^. Canonical β-catenin mediated Wnt signaling directly regulates osteoblast differentiation and activity and likely has indirect effects on osteoclasts during bone metabolism^4^. Moreover, a study reported the direct negative influence of canonical Wnt/β-catenin signaling on osteoclast development using in vitro cell models and in vivo mouse studies^5^. During embryogenesis, canonical Wnt/β-catenin signaling is highly active in the first pharyngeal arch as well as in multiple craniofacial regions in mouse, chicken and zebrafish^6–8^. Wnt signaling is involved in regulating skeletogenic neural crest cells, such as the subdivision of each pharyngeal arch into dorsal and ventral elements in zebrafish during craniofacial development^9^. In addition, Wnt signaling plays a role in mediating regional specification in the vertebrate face^7^. The identification of modulators of Wnt signaling during development and homeostasis of adult skeletal tissues may lead to new insights into disease etiology and identify potential targets for therapeutic mediation.

Human genome wide association studies revealed many regulators of canonical Wnt signaling that are involved in regulating bone metabolism^10,11^. The *R-spondin* (*Rspo*) family of secreted proteins includes four members (*Rspo1*-*4*) in the thrombospondin type 1 repeat (TSR1)-containing protein superfamily that have been shown to potentiate the canonical Wnt/β-catenin pathway^12,13^. RSPO proteins modulate Wnt signaling through interactions with the LGR4-6 receptors, leading to stabilization of Frizzled and LRP5/6 at the cell membrane, and through regulation of the ubiquitin ligases ZNFR3 and RNF43 that degrade Frizzled receptors^13,14^. *Rspo2* and *Rspo3* also have been shown to augment Wnt/β-catenin signaling independent of LGRs by binding to heparin sulfate proteoglycans^15^. *Rspo* genes are essential for normal development and have been shown to regulate skeletal patterning during development^16^. In particular, *Rspo2* has been shown to be essential for limb patterning^17^. Additionally, several GWAS conducted in humans have associated *RSPO2* and *RSPO3* with bone mineral density^10,18^.

*Rspo3* was identified as a candidate gene that contributes to cleft lip/palate and dental anomalies^19^. *Rspo3* was also reported to have a critical role in mouse placental development^17^. However, since mouse embryos lacking *Rspo3* function die at E10.5 due to placenta and vascular defects, this precluded analysis of its role during later embryonic development^17^. Conditional ablation of the *Rspo3* in limb mesenchymal cells caused modest delay in limb growth during development^20^. *Rspo3* and *Rspo2* double mutant mice however developed severe hindlimb truncations, suggesting a redundant function of these genes^20^. The function of *Rspo3* during craniofacial morphogenesis has yet to be defined^21^.

Wnt/β-catenin pathway also plays a critical role in tooth development and can affect craniofacial development more broadly^22,23^. Tooth formation initiates from the interactions between the dental epithelial layer and the underlying mesenchyme^24,25^. Mice have a single set of dentition (monophyodont) that consists of continuously erupting incisors, and three molars in single row on both sides of the upper and lower jaws that do not exhibit continuous growth or replacement^26,27^. The zebrafish dentition is more numerous, unlike in the mouse, zebrafish teeth exhibit continuous replacement throughout life (polyphyodont)^28^. Despite these differences, the molecular and cellular mechanisms regulating tooth development are highly conserved between zebrafish and mammals^28^. Therefore, studies in zebrafish can provide novel insights into the regulation of craniofacial structures that can complement the mouse.

Here, we focused on the roles of *Rspo2* and *Rspo3* in regulating dental and craniofacial development. We utilized RNAscope probes to gain high resolution images of *Rspo2 and Rspo3* gene expression in zebrafish and mouse. We examined the genetic requirement of *rspo2 and rspo3* in zebrafish development, using complementary CRISPR/Cas9-mediated targeted mutagenesis. Using these approaches, we revealed roles for r*spo2 and rspo3* in tooth development and in morphogenesis of the craniofacial complex.

## Results

### *Rspo2* and *Rspo3* are expressed in the craniofacial complex and in the perichondrium and osteoprogenitor cells during zebrafish craniofacial morphogenesis

**Figure 1:**
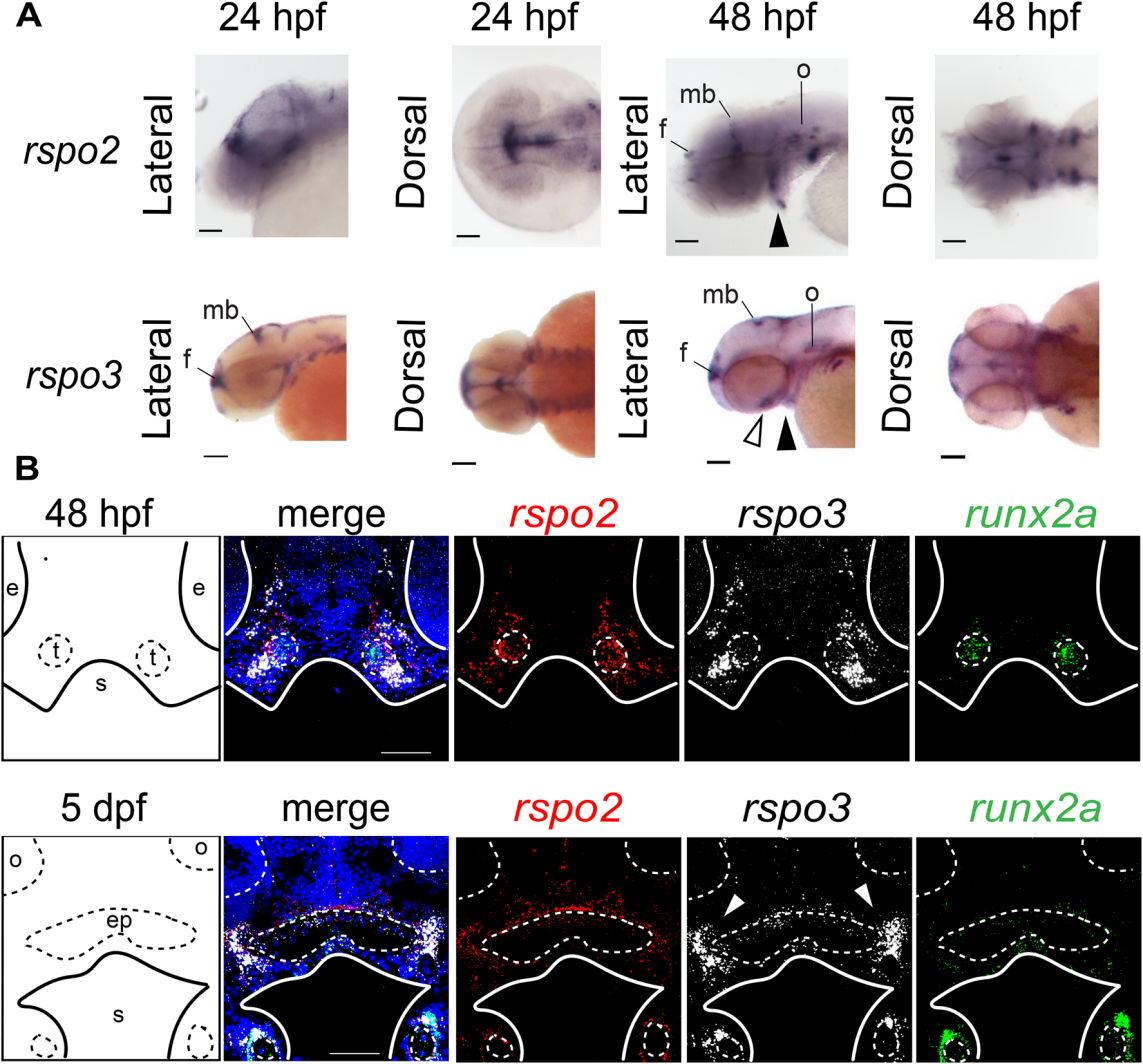
High resolution gene expression analysis detected dynamic spatiotemporal localization of *rspo2* and *rspo3* transcripts in zebrafish cranial mesenchyme. (**A**) Whole-mount RNA in situ hybridization detected *rspo2* and *rspo3* transcripts in the midbrain (mb), forebrain (f), otic vesicle (o), and Meckel’s cartilage (arrowhead) at 24 and 48 hpf in lateral and dorsal views. Transcript of *rspo3* was additionally detected at 24 and 48 hpf in the forebrain (f) and at 48 hpf in the ethmoid plate (open arrowhead). Scale bar: 100 um. (**B**) Maximum projections of z-stacks of coronal sections of zebrafish embryos, section in plane with eyes. Labeled schematic at left. At 48 hpf, *rspo2* and *rspo3* transcripts were highly co-localized in the mesenchyme surrounding the condensing trabeculae cartilage mesenchyme, marked by *runx2a* expression. *rspo2* is also detected within the condensing mesenchyme at this timepoint. At 5 dpf *rspo2* and *rspo3* continue to be co-expressed in mesenchyme and perichondrium surrounding cartilage elements, specifically the ethmoid plate and Meckel’s cartilage. *Rspo3* expression is particularly high in *runx2a* expressing osteogenic precursor cells associated with Meckel’s cartilage, as well as where the palatoquadrate meets the ethmoid plate (white arrowhead). Abbreviations: e: eye, o: olfactory organ, s: stomodeum, t: trabeculae. Scale bar: 100 μm.

Gene expression patterns of *rspo2* and *rspo3* during zebrafish embryogenesis were delineated by whole-mount RNA in situ hybridization (WISH). *rspo2* and *rspo3* transcripts were detected in the brain, otic vesicle, and endodermal pouches at 24 h post-fertilization (hpf) and as well as in regions consistent with the ethmoid plate and Meckel’s cartilage at 48 hpf (Fig. 1A). Using RNAscope *in situ* hybridization, we identified diffuse *rspo3* transcript expression throughout the mesenchyme with concentrated expression in cells that circumscribe the pre-cartilage mesenchyme (48 hpf) and the paired trabeculae, ethmoid plate, and Meckel’s cartilage at 5 days post-fertilization (dpf) (Fig. 1B). We also detected *rspo3* expression within ethmoid plate chondrocytes at 5 dpf (Fig. 1B). *rspo2* expression generally overlapped with *rspo3* at both developmental timepoints.

### Expression of *rspo3* is similar in mouse and zebrafish, however* rspo2* expression is distinct

**Figure 2:**
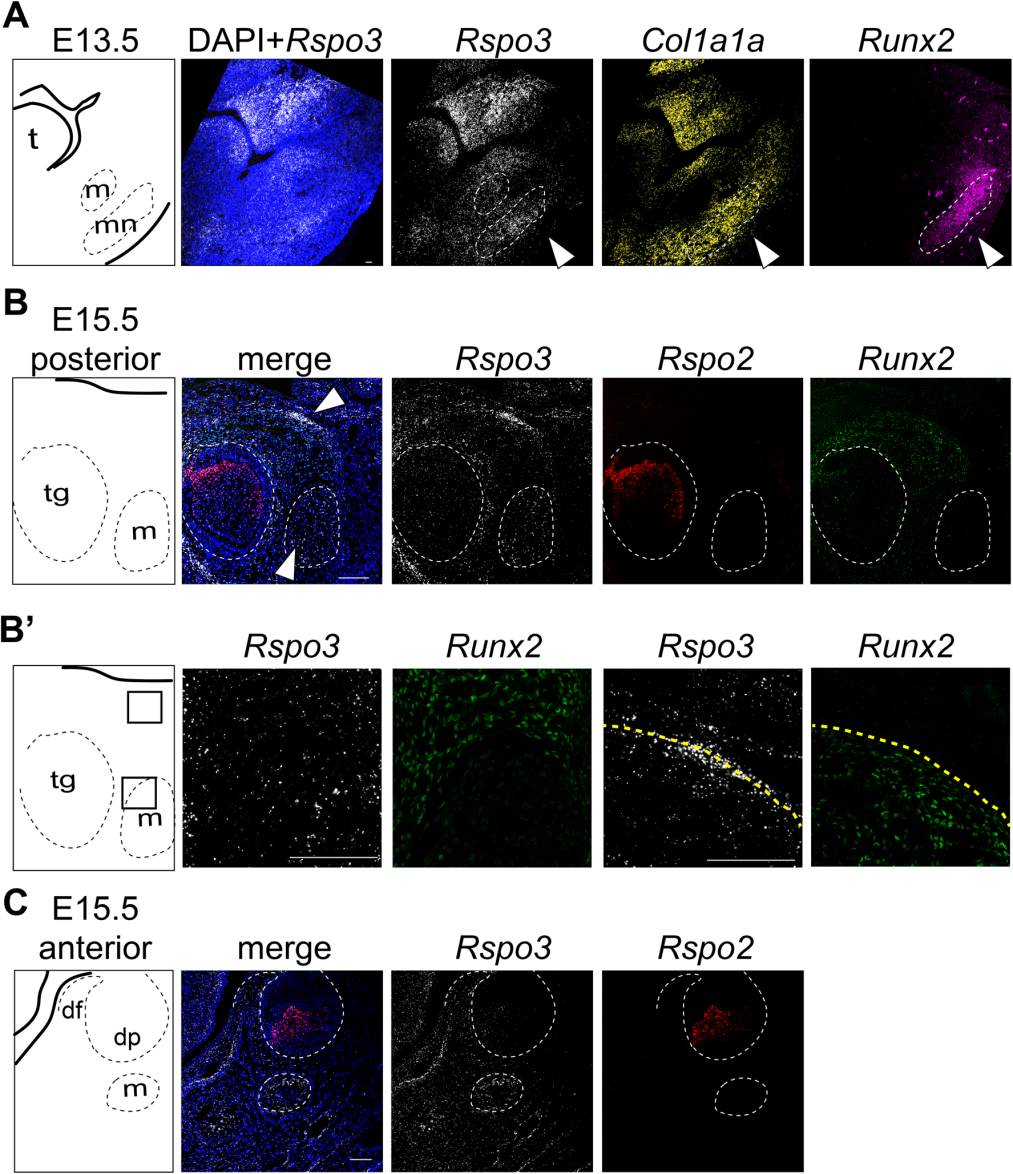
RNAscope gene expression analysis of *Rspo2* and *Rspo3* in mouse embryos. Coronal sections of wild-type mouse embryos at E13.5 and E15.5. Labeled schematic at left. (**A**) RNAscope revealed diffuse expression of *Rspo3* throughout the cranial mesenchyme at E13.5. *Col1a1 *in situ hybridization and *Runx2* immunofluorescence were used to identify osteogenic precursor cells and *Rspo3* expression was detected within these cells. Of particular note is *Rspo3* expression within Meckel’s cartilage (m) and the developing mandible (arrow; mn). (**B**) At E15.5, *Rspo3* expression is detected diffusely thorough the mesenchyme, including in Meckel’s cartilage (m) and within osteogenic precursor cells (co-expressed with *Runx2*). *Rspo2* expression is isolated to discrete cells within the developing tooth germ (tg). (**B’**) Higher magnification images (boxes depict location) of *Rspo3* and *Runx2* co-expression. (**C**) Within the developing molar at E15.5, *Rspo2* and *Rspo3* transcripts were detected in distinct non-overlapping regions, with *Rspo3* expression in Meckel’s cartilage (m) and the dental follicle (df), while *Rspo2* is expressed exclusively within the dental pulp (dp). Scale bar: 100 μm.

To test the conservation of *Rspo2* and *Rspo3* expression between vertebrates, we analyzed expression in mouse embryos with RNAscope in situ hybridization and immunofluorescence. At E13.5 we detected *Rspo3* expression in regions consistent with osteogenesis, including the developing mandible. We found cellular co-localization of *Rspo3*, C*ol1a1* mRNA and *Runx2* protein, indicating a potential role in osteogenesis (Fig. 2A). In the E15.5 mouse embryo, *Rspo3* transcripts were detected widely throughout the mesenchyme as well as within Meckel’s cartilage (Fig. 2B). *Rspo3* expression was also detected within *Runx2* positive, presumptive osteoprogenitor cells (Fig. 2B). In contrast to gene expression results in zebrafish, we did not detect *Rspo2* expression in the mesenchyme of mouse embryos or associated with cartilage elements. Instead, within the developing mandible, *Rspo2* expression was restricted to developing teeth (Fig. 2C). Unlike in zebrafish where *rspo2* and *rspo3* expression largely overlap, in the mouse transcripts of *Rspo2* and *Rspo3* appear to be anatomically distinct (Fig. 2).

### *rspo2* and *rspo3* are differentially expressed within zebrafish dental structures

**Figure 3:**
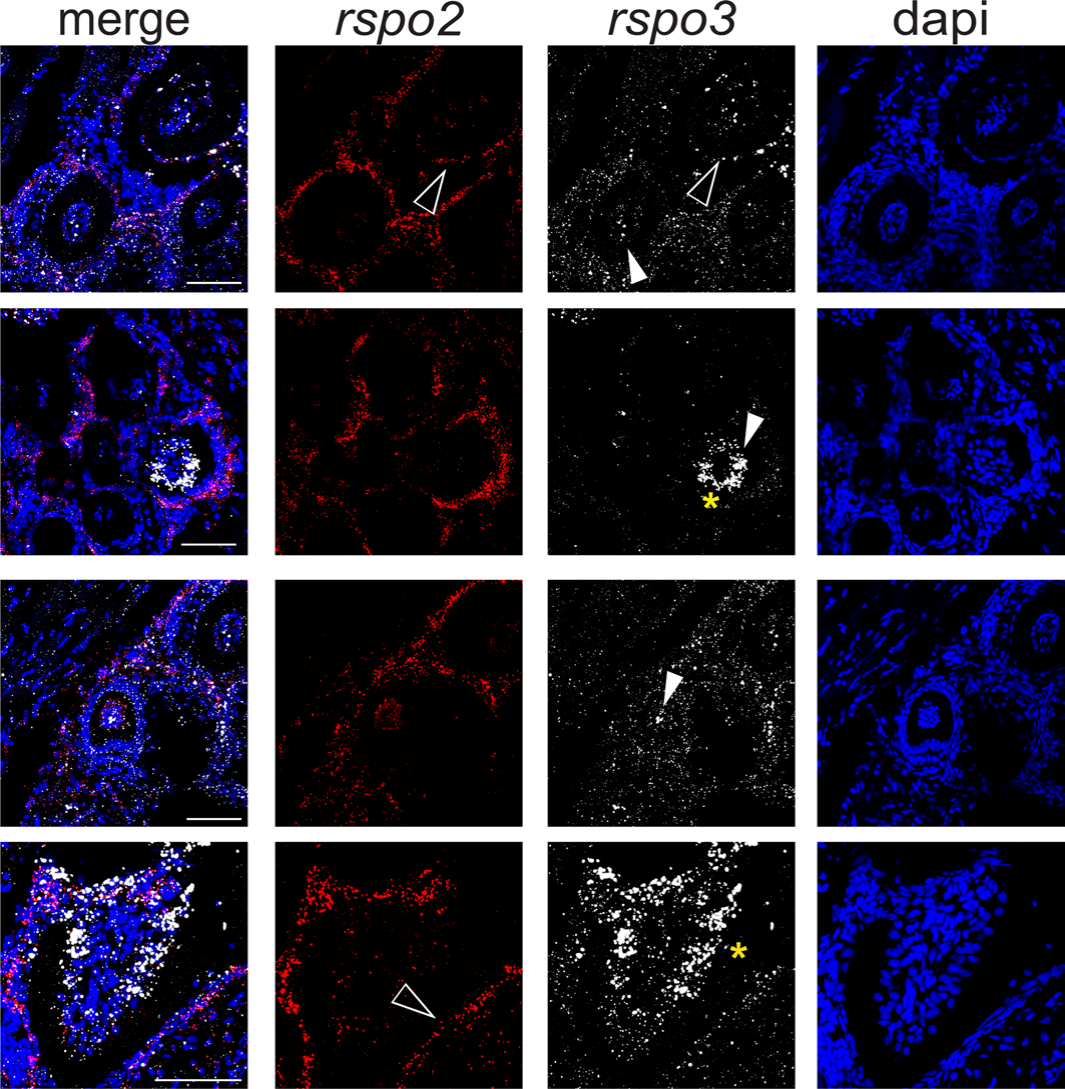
*rspo2* and *rspo3* are differentially expressed in zebrafish pharyngeal teeth. Representative images of maximum projections of z-stacks of sagittal sections of 180 dpf zebrafish. *rspo2* (red) and *rspo3* (white) transcripts were detected by RNAscope in situ hybridization. *rspo3* is diffusely expressed throughout the supporting mesenchyme and highly expressed in enamel epithelium (open arrow) and some tooth pulp (closed arrow). Often within the tooth pulp, *rspo3* expression is restricted to the outermost odontoblasts (*). Meanwhile, *rspo2* expression is highest in enamel epithelium (open arrow) and very low within the tooth pulp. Scale bar: 100 μm.

Given the expression of *Rspo2* and *Rspo3* in developing mouse teeth, we examined the gene expression of *rspo2* and *rspo3* within and surrounding the tooth structure in zebrafish. *rspo3* gene expression was detected at low levels diffusely throughout the dental pulp and the surrounding mesenchyme (Fig. 3). In contrast, high levels of *rspo2* gene expression were detected in the enamel epithelium (Fig. 3). Furthermore, *rspo3* gene expression was highest within odontoblasts of regenerating teeth (Fig.3).

### Combined disruption of *rspo2 and rspo3* resulted in cartilage dysmorphogenesis

Given the specific expression of *rspo3* in early palate and in Meckel’s cartilage development, we used CRISPR/Cas9-mediated genome editing to generate *rspo3* mutant alleles. Guide RNAs targeting the *rspo3* gene in exon2 were used to create a *rspo3* mutant germline allele (Fig. S1A). A frame shift mutation was generated by introducing a − 20 bp gene deletion, as detected by microsatellite genotyping and confirmed by Sanger sequencing (Fig. S1B). The efficiency of this *rspo3*–20 bp deletion allele (hereafter called *rspo3*^−/−^) was assessed by qRT-PCR at 6 hpf, where we observed that *rspo3* mRNA was significantly reduced by sixfold in the mutant compared as compared to wild-type clutch-mates (p < 0.05; Fig. S1C).

**Figure 4:**
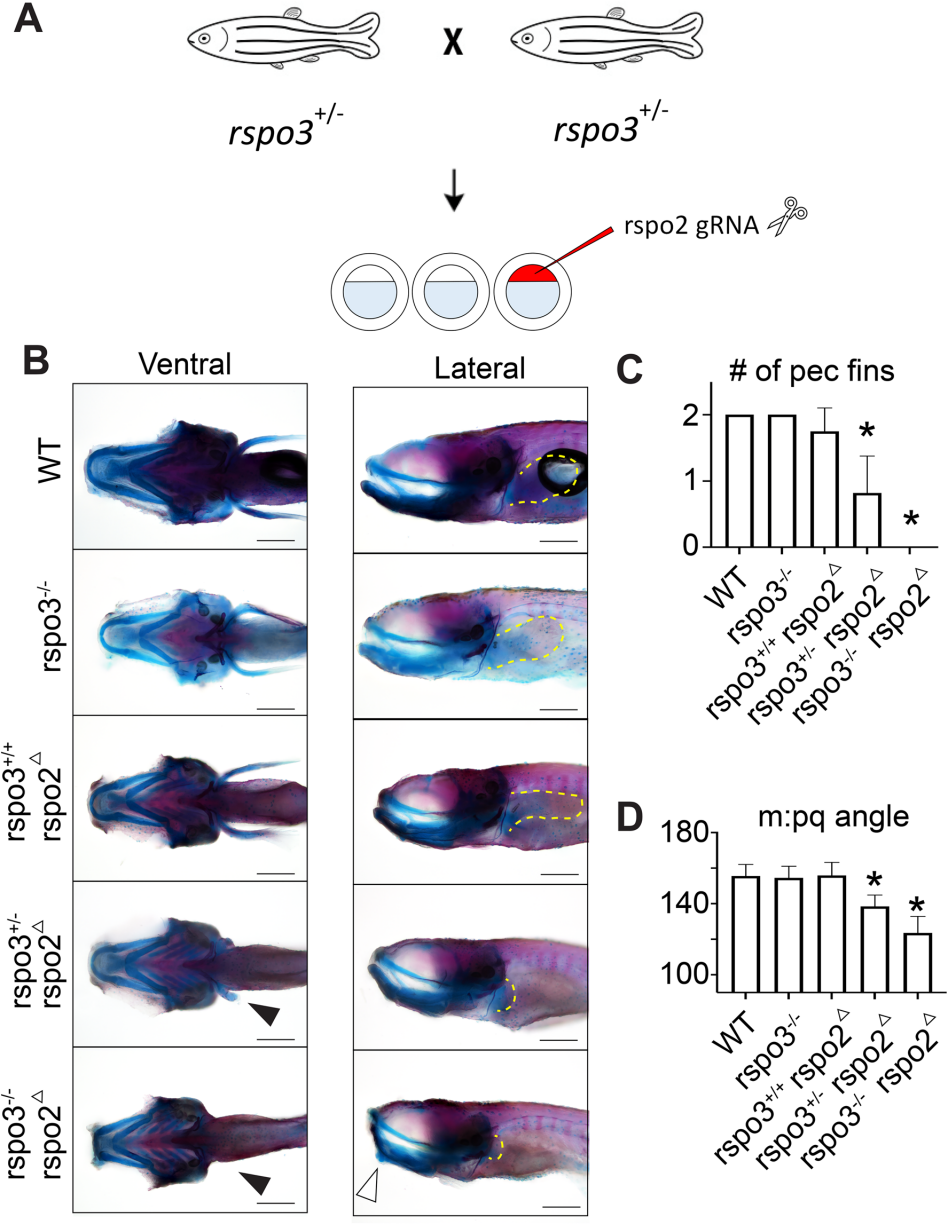
Synergistic effect of *rspo2* and *rspo3* ablation on zebrafish limb development and craniofacial morphology. (**A**) Schematic illustrating experimental design. Targeted mutagenesis of *rspo3*^−/−^ in zebrafish was carried out using CRISPR/Cas9 gene editing. A – 20 bp deletion was bred to homozygosity. Intercross or *rspo3*^+/−^ were injected with 4 gRNAs against *rspo2* and the resulting larvae were genotyped and analyzed for phenotype. (**B**) Whole mount ventral and lateral images of Alcian blue/Alizarin red S stained 9 dpf larvae. *rspo3*^−/−^ embryos that were *rspo2* gRNA/Cas9 injected (*rspo2*^Δ^) larvae were similar to wild-type except that *rspo2*^Δ^ larvae exhibited disrupted development of the pectoral fin. Impaired fin development was exacerbated with decreasing genetic dosage of *rspo3* (black arrows, dotted yellow lines delineate fins). While craniofacial development in *rspo3*^−/−^ and *rspo2*^Δ^ larvae were largely normal, *rspo3*^−/−^; *rspo2*^Δ^ double mutants exhibited a dysmorphic lower jaw (white arrow). Scale bar: 100 μm. (**C**) Quantification of pectoral fin developmental disruption. *rspo2*^Δ^ larvae tended to have disrupted development of a single pectoral fin. This effect was significantly exacerbated with decreasing genetic dosage of *rspo3*, as *rspo3*^−/−^; *rspo2*^Δ^ double mutant larvae failed to develop pectoral fins altogether. (**D**) Quantification of angle measurements between Meckel’s cartilage (m) and palatoquadrate (pq). While *rspo3*^−/−^ and *rspo2*^Δ^ mutants had normal lower jaw morphology, rspo3^+/−^; *rspo2*^Δ^ and rspo3^−/−^; *rspo2*^Δ^ mutants displayed a significantly decreased angle at the Meckel’s/palatoquadrate joint. N = 10–16. p < 0.01. *Indicates significance relative to wild-type.

To characterize requirements for *rspo3* during early craniofacial morphogenesis, Alcian blue cartilage staining was performed at 5 dpf. The effects of *rspo3* disruption on larval cartilage skeleton were found to be subtle. As *rspo2* is known to also function in regulating Wnt signaling and has overlapping expression in the zebrafish, we hypothesized that *rspo2* action may be compensating for *rspo3* germline disruption. Therefore, to determine the combined requirement of *rspo2* and *rspo3*, we targeted *rspo2* by injection of multiple gRNA into *rspo3* homozygous embryos (Fig. 4A), commonly referred to as a crispant and denoted here as *rspo2*^Δ^^29^. Embryos generated from *rspo3*^+/−^ in-crossed zebrafish were either raised for analysis of the single mutant or were injected at the 1-cell stage with gRNAs targeting *rspo2* (*rspo2*^Δ^). The resulting larvae were stained at 9 dpf with Alcian blue and Alizarin Red S. Following imaging and phenotyping, individual larvae were genotyped. We identified a subset of zebrafish with disrupted pectoral fin development where either the fin was partially formed or was absent (Fig. 4B). We found that *rspo2* was required for pectoral fin development, and that haploinsufficiency of *rspo3* exacerbated the loss of pectoral fin formation (Fig. 4B,C).

In addition to altered fin development, we identified a subset of zebrafish with altered craniofacial morphology affecting the lower jaw. We found that *rspo2*^Δ^, *rspo3*^+/−^ and *rspo3*^−/−^; *rspo2*^Δ^ larvae displayed a significantly reduced angle where the palatoquadrate meets Meckel’s cartilage (Fig. 4B,D).

To evaluate craniofacial effects in greater detail and visualize individual cartilage elements, we dissected out the ethmoid plate and ventral cartilages, including the pharyngeal teeth (Fig. 4). Analyses of Alcian blue/Alizarin Red S zebrafish at 9 dpf revealed that disruption of *rspo2* caused a decrease in the number of pharyngeal teeth, with an average of 2 total teeth rather than the 8 teeth observed in the control (Fig. 4B). Although *rspo3*^−/−^ larvae did not exhibit a difference in the number of teeth at 9 dpf, haploinsufficiency of *rspo3* decreased tooth number in the *rspo2*^Δ^ larvae, with the *rspo3*^−/−^; *rspo2*^Δ^ double mutant having no mineralized teeth at 9 dpf (Fig. 4A,B).

**Figure 5:**
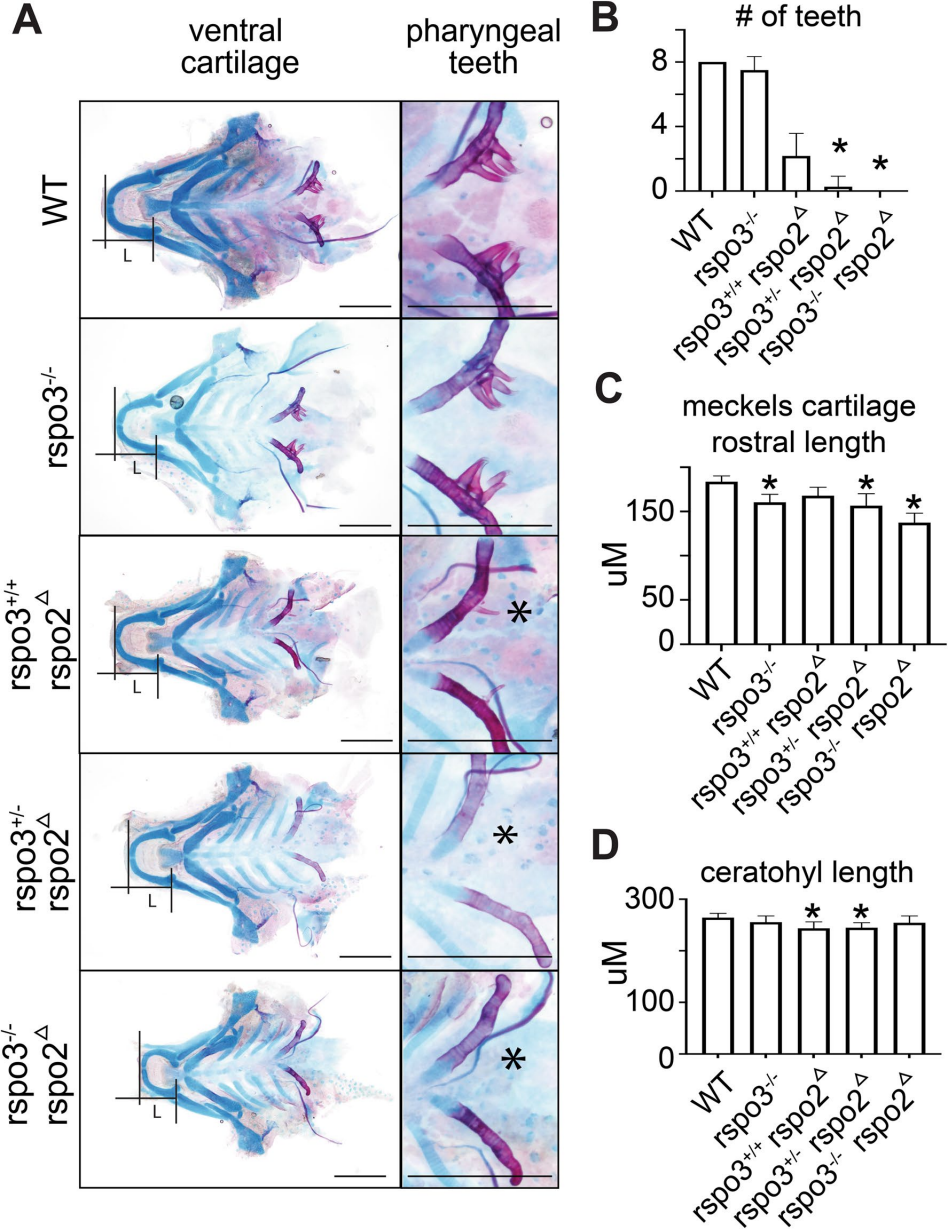
Synergistic effect of *rspo2* and *rspo3* ablation on zebrafish tooth development and Meckel’s cartilage. (**A**) Flat-mount images of Alcian blue/Alizarin red S stained 9 dpf zebrafish ventral cartilages. Zoom of pharyngeal teeth to right. *rspo3*^−/−^ larvae displayed anterior shortening of Meckel’s cartilage, which was exacerbated with *rspo3*^−/−^; *rspo2*^Δ^ gRNA disruption (black bars). *indicate absent teeth. Scale bar: 200 μm. (**B**) Alizarin red S staining of pharyngeal teeth shows that *rspo3*^−/−^ are generally normal relative to wild-type while *rspo2*^Δ^ larvae have a reduced number of teeth (average of 2 versus 8). Tooth number in *rspo2*^Δ^ larvae decreased further with decreasing wild-type alleles of *rspo3* (zero teeth detected in *rspo3*^−/−^; *rspo2*^Δ^ mutant). (**C**) Quantification of the anterior–posterior/rostral length of Meckel’s cartilage shows a primary effect in *rspo3*^−/−^ larvae, which is exacerbated in *rspo3*^−/−^; *rspo2*^Δ^ mutants. (**D**) Quantification of the anterior–posterior length of ceratohyal cartilage shows no effect in rspo3^−/−^ larvae, suggesting a cartilage element-specific effect of *rspo3* and *rspo2*. N = 10–16. p < 0.01. *Indicates significance relative to wild-type.

Flat-mount imaging of Alcian blue/Alizarin Red S-stained ventral cartilage revealed a significant decrease in anterior–posterior/rostral length of Meckel’s cartilage in the *rspo3*^−/−^ larvae while *rspo2* disruption alone had no effect (Fig. 5A,C). The requirement for *rspo3* on Meckel’s cartilage rostral length was significantly exacerbated by *rspo2* disruption (Fig. 5C). The effect of *rspo3* on Meckel’s cartilage rostral length is specific, rather than due to a total anterior–posterior shortening, as ceratohyal length anterior–posterior length was not different in these zebrafish (Fig. 5D).

### *rspo3* influences osteoclast activity during zebrafish development

**Figure 6:**
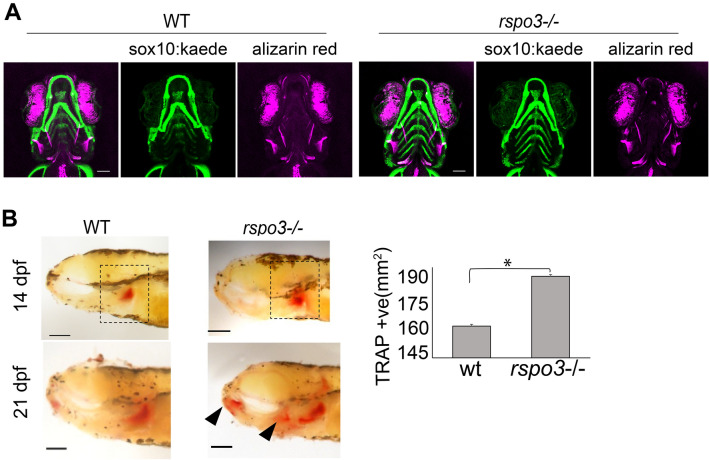
*rspo3* ablation does not impact initial bone mineralization but increases osteoclast area of activity. (**A**) Representative images of maximum intensity projections of confocal z-stack of whole-mount 10 dpf zebrafish. Zebrafish express *sox10:kaede* transgene allowing fluorescent imaging of cartilage elements. Zebrafish were live-stained with Alizarin red S to visualize mineralized structures. No differences in intensity of Alizarin staining, elements stained, or cartilage architecture were noted in the *rspo3*^−/−^ fish. Scale bar: 100 μm. (**B**) Lateral view of 14 dpf showing increased osteoclast activity (red stain in the black dotted box) in *rspo3* mutant as compared to wild-type. At 21 dpf, more areas of osteoclast activity in the dentary, hyomandibular, pharyngeal teeth and jaws (solid arrow) were observed in *rspo3*^−/−^ compared to wild-type. Quantification of total area of red staining. P < 0.05. N = 5. Scale bar: 100 μm.

To assess the role of *rspo3* on osteogenesis in developing zebrafish we performed live Alizarin Red S staining on 10 dpf *rspo3*^+/+^ and *rspo3*^−/−^ larvae. The *rspo3* mutant allele was also bred onto a *sox10:kaede* background in order to visualize cartilage elements. Confocal analyses of whole mount embryos revealed no differences in Alizarin Red S intensity (Fig. 6A). No obvious changes in cartilage morphology were observed in the *rspo3*^-/-^ fish. Interestingly, we did observe increased tartrate-resistant acid phosphatase (TRAP) positive area in *rspo3*^*-/-*^ mutants at 14 and 21 dpf, suggesting increased osteoclast number (Fig. 6B). Therefore, these results indicate that *rspo3* may regulate aspects of bone homeostasis after larval development as the animals mature during adult life.

### Adult rspo3 zebrafish mutants have decreased body length and exhibit a midface deficiency

**Figure 7:**
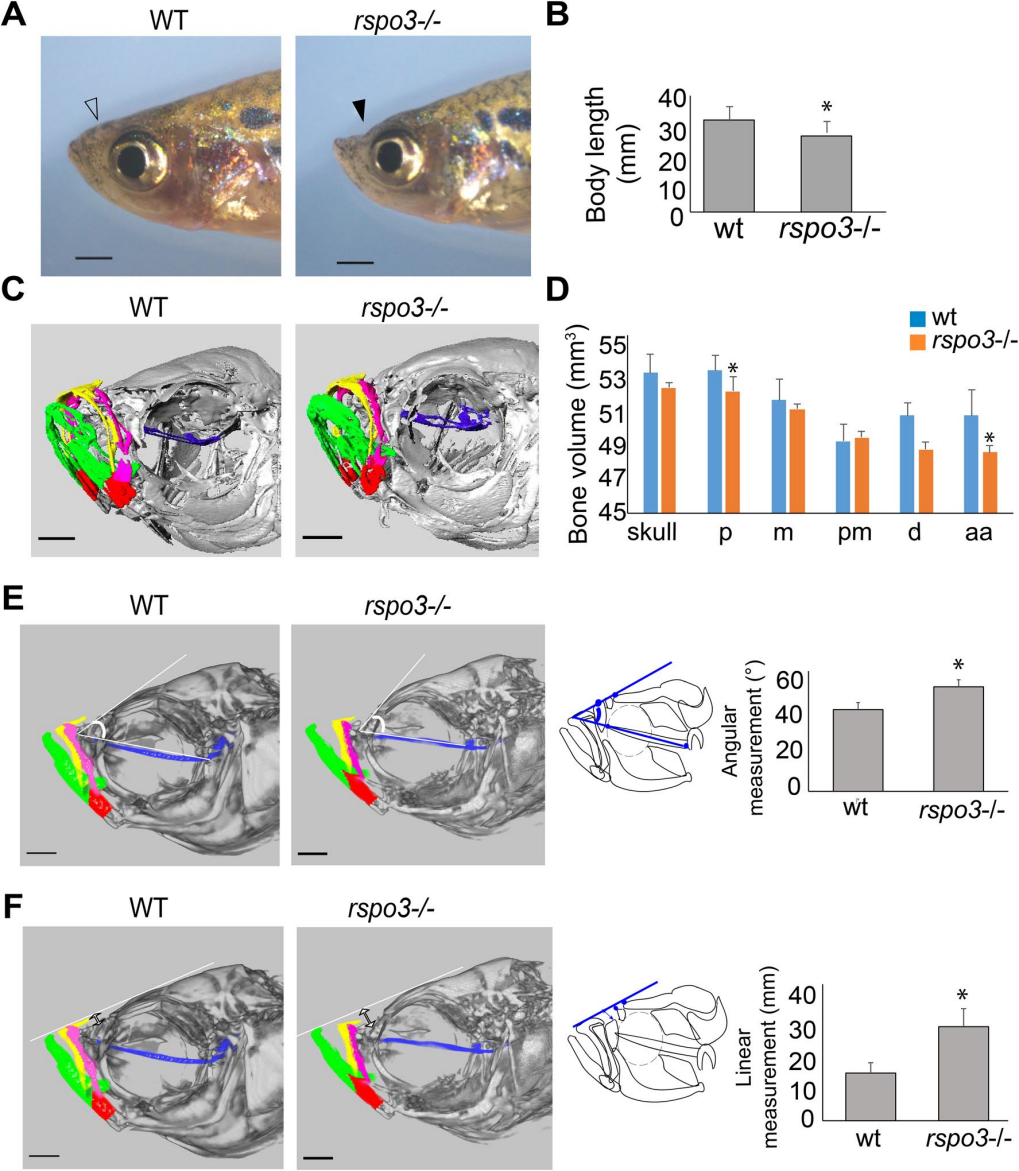
*rspo3* mutants exhibited midface deficiency, frontal bossing and decreased body length. Reduced body length, midface deficiency and frontal bossing were observed in adult *rspo3*^−/−^ (180 dpf). (**A**) Lateral image of adult zebrafish showing midface depression in *rspo3*^−/−^ (solid arrow) compared to wild-type (open arrow). (**B**) Body length was significantly decreased in *rspo3*^−/−^ mutants relative to wild-type. (**C**) Oblique micro-CT image of *rspo3*^−/−^ and wild-type fish at 180 dpf. Individual bone elements are color coded (blue: parashenoid, pink: maxilla, yellow: premaxilla, green: dentary and red: anguloarticular). Scale bar: 10 μm. (**D**) Bone volume of the skull and of specific bones in *rspo3*^−/−^ and wild-type individuals demonstrates element-specific differences in volume. Parasphenoid and anguloarticular bone volume were significantly reduced in *rspo3*^−/−^ compared to wild-type fish. Abbreviations: aa: anguloarticular, d: dentary, m: maxilla, p: parasphenoid, pm: premaxilla. (**E**) 2D cephalometric analysis obtained from micro-CT of *rspo3*^−/−^ and wild-type fish. The angle formed by parasphenoid line and a line tangent to frontal bone identified frontal bossing, with increased angle in *rspo3*^−/−^ compared to wild-type. Diagram of lateral view of adult zebrafish showing the angular measurement. Bar chart showing statistical differences in the angular measurement between *rspo3*^−/−^ and wild-type. (**F**) 2D cephalometric analysis of *rspo3*^−/−^ and wild-type fish. The distance between nasal bone and a line drawn between dentary and frontal bone landmarks were measured. Diagram of lateral view of adult zebrafish showing the linear measurement from nasal bone to a line tangent to the frontal bone and dentary. The linear measurement value was significantly greater in *rspo3*^−/−^ mutants than in wild-type indicating the presence of midface hypoplasia. *p ≤ 0.05*.* Scale bar: 100 μm.

As *rspo3*^−/−^ mutant zebrafish larvae matured to adult fish, we observed midface hypoplasia compared to wild-type clutch-mates (Fig. 7A). Statistically significant differences in body length (measured from tip of mouth opening to the base of the tail, STL) were observed in *rspo3*^−/−^ mutant as compared to wild-type clutch-mates (Fig. 7B). In addition, *rspo3*^−/−^ mutant exhibited significantly decreased parasphenoid and anguloarticular bone volume compared to wild-type clutch-mates (Fig. 7C, D). The altered morphology of individual bony elements in *rspo3*^−/−^ zebrafish also resulted in altered relationships between the bony elements. Cephalometric analysis revealed significant frontal bossing in *rspo3*^−/−^ mutant adults, with increased parasphenoid-frontal angle (Fig. 7E). Furthermore, we observed midface hypoplasia in adult *rspo3*^−/−^ zebrafish as compared to wild-type, with significant increased distance between nasal bone and a line drawn between dentary and frontal bone landmarks (Fig. 7F and Supplemental Videos S1, S2).

### *rspo3* is required for normal tooth maintenance

**Figure 8:**
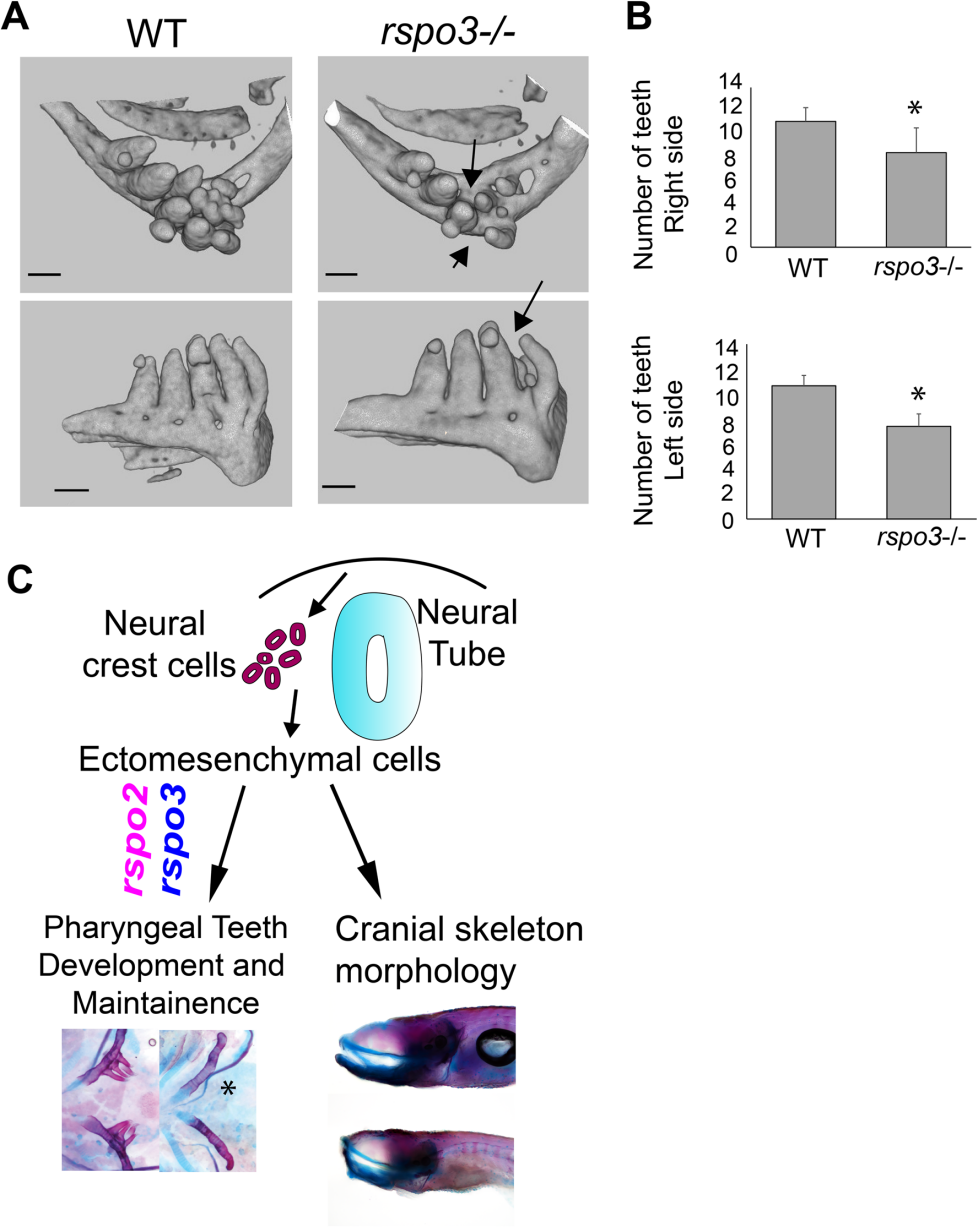
Adult *rspo3* mutant zebrafish have reduced teeth number. (**A**) Micro-CT of 180 dpf zebrafish reveals that the adult *rspo3*^−/−^ animals exhibit decreased tooth number with several sockets missing teeth that are present in the wild-type. (**B**) *rspo3*^−/−^ have significantly fewer teeth than wild-type on both right and left sides. N = 9.7. *p < 0.05. (**C**) Summary diagram illustrating that *rspo2* and *rspo3* both regulate pharyngeal tooth development as well as have roles in morphogenesis of the craniofacial skeleton.

Analysis of pharyngeal tooth morphology in adult zebrafish using micro-CT illustrated decreased tooth number in *rspo3*^−/−^ mutant zebrafish, as compared to wild-type clutch-mates (Fig. 8A,B). On average, *rspo3*^−/−^ adult zebrafish had two fewer teeth on both the right and left sides of the jaw (Fig. 8B). As we found no difference in the number of teeth during the larval stage in *rspo3*^−/−^ animals (Fig. 5), we suggest that *rspo3* functions in the maintenance of teeth, rather than tooth development, either by regulating tooth integrity or regulating tooth regeneration.

## Discussion

This study reports *Rspo2* and *Rspo3* gene expressions and functions in craniofacial and dental morphogenesis, using zebrafish and mouse models. *Rspo3* is diffusely expressed through the craniofacial mesenchyme whereas *Rspo2* is expressed in distinct domains. In zebrafish, there is overlap in *rspo2* and *rspo3* gene expressions, whereas in the mouse embryo the expression domains of these paralogs are distinct. We showed that *Rspo3* is expressed in perichondral cells, and *Runx2* positive osteoprogenitors in embryonic palate and Meckel’s cartilage in zebrafish, as well as in mouse osteoprogenitors. In zebrafish teeth, *rspo3* is expressed in newly formed replacement teeth, where it is broadly expressed in dental pulp, odontoblasts, and crypt epithelium. Analysis of adult *rspo3*^−/−^ zebrafish suggest that *rspo3* is required for adult teeth maintenance. Loss of *rspo3* did not affect larval osteogenesis but did result in increased area of TRAP staining, midface hypoplasia, and reduced numbers of attached teeth in adult zebrafish. Importantly, *rspo2* and *rspo3* genetically interact, where haploinsufficiency of *rspo3* exacerbates defects in tooth formation and pectoral fin bud extension. We show that zebrafish *rspo2* and *rspo3* are required for limb development, analogous to the mouse function of *Rspo2/3* that was previously reported^20^. Together, these gene expression studies and genetic analyses are consistent with functions for *rspo3* in progenitor cell populations contributing to the craniofacial skeleton and teeth, and in the maintenance of craniofacial bones and teeth in zebrafish (Fig. 8C).

We showed that high resolution gene expression analysis using RNAscope on sectioned specimens provided greater resolution as compared to whole mount (ISH) approaches (compare Fig. 1A,B). Using RNAscope, we were able to determine that *rspo2* and *rspo3* transcripts were detected in a cell layer that surrounds the chondrogenic elements in the zebrafish anterior neurocranium, trabeculae and in Meckel’s cartilage. Moreover, *Rspo3* is co-expressed with *Runx2* (osteoprogenitor marker) in zebrafish and mouse (Figs. 1, 2). In support of our findings, a human genetic study recently reported the involvement of *RSPO3* in bone mineral density and bone fractures^30^. Furthermore, *RSPO3* was reported to regulate osteoblastic differentiation^31^. In addition, human *RSPO3* was identified as a candidate gene that contributes to cleft lip/palate and dental anomalies, consistent with its role in skeletal development and in human adipose-derived stem cells^19,31^. Taken together, this and other studies corroborate that *Rspo3* has conserved functions in the development of craniofacial bone and tooth structures across vertebrates.

This study also identified a key requirement for *rspo3* in regulating tooth development. Zebrafish teeth are continuously replaced through its life, where the regenerative process is analogous to human adult tooth replacement of a deciduous, baby tooth and to mouse continuously growing incisors^28,32,33^. We described *rspo3* gene expression in dental pulp, odontoblasts and crypt and dental epithelium in zebrafish and mouse, suggesting possible roles in the regulation of tooth development, odontogenesis and ameloblast differentiation. Importantly, *rspo3* is highly detected in zebrafish replacement teeth as compared to mature teeth, indicating potential roles in dental progenitor cell populations as compared to more differentiated dental cell types. Moreover, adult *rspo3*^−/−^ zebrafish exhibit reduced attached tooth numbers as compared to age-matched wild-type fish. The normal tooth formation in *rspo3*^−/−^ mutant zebrafish at 9 dpf while having reduced tooth number at 180 dpf suggests a role for rspo3 in the maintenance of adult teeth rather than in their initial development. Differences in *Rspo2/3* spatiotemporal gene expression in mouse and zebrafish may reflect differences in the regenerative odontogenic potential of zebrafish. Wnt/β- catenin signaling is important for tooth morphogenesis, and consistent with the observation that *rspo3* disruption resulted in inhibited dental tissue development^25^.

Adult *rspo3*^−/−^ zebrafish exhibited midface hypoplasia, frontal bossing and reduced tooth number as compared to aged-matched wild-type clutch-mates. Our data showed co-expression of *rspo3* and *col1a1a* during embryogenesis, suggesting that these two genes could be functionally associated. Consistent with this result, previous studies reported that patients with osteogenesis imperfecta have mutations in *COL1A1A* which is characterized by frontal bossing, midface hypoplasia and dentinogenesis imperfecta^35–37^. Future studies are recommended to investigate the molecular mechanisms regulated by *rspo3*, including its interactions with Wnt signaling pathway genes in regulating dental and bone development.

## Methods

### Experimental animals

All animal experiments were approved by the Massachusetts General Hospital (MGH) Institutional Animal Care and Use Committee (IACUC) and in compliance with ARRIVE guidelines. Zebrafish embryos and adults were cared for and maintained in this study as previously described^38^. Wild-type mice were ordered obtained from Jackson Laboratory (C57BL/6J, Bar Harbor, ME, USA) and *Rspo3* mutant mice were kindly provided to Dr. Baron by Dr. Christof Nierhs (German Cancer Research Center, Heidelberg, Germany). All methods were carried out in accordance with relevant guidelines and regulations.

### Zebrafish CRISPR mutant line, F0 CRISPR disruption and reporter lines

We used targeted genome editing via CRISPR-cas9 mutagenesis in zebrafish to perform functional analysis of *rspo3*. A *rspo3* mutant zebrafish line was created using the cas9 RNA CCTGGCAGCCCTGGGAGCTC, which resulted in a 20 bp deletion (Supplemental Fig. S1). Genotyping primers for the *rspo3* mutant line are 5′-AAGCAGCAAAAATAAGTTCCCA-3′ and 5′-CCACTCCCCATTGCTTTATTAC-3′ with FAM modification on the reverse primer for microsatellite analysis. The mutant peak was observed at 337 bp and wild-type peak observed at 357 bp.

CRISPR gRNA were designed using CRISPOR (http://crispor.tefor.net/) to target *rspo2* translational start sites as previously described^29^. Due to the presence of two *rspo2* transcript variants with unique translational start sites (TSS), specific pairs of gRNAs (4 total) were designed to flank each TSS. Guides ordered from Synthego were the following: AGCTCATATACGGACCCTGAAGG, AGACGCAGCAGTCCCACCGCTGG, ATGTCTTTGTACCAAACGATTGG, TCCTCTCCCTCCTCAGGAACAGG.

All four gRNAs were co-injected into *rspo3*^+/−^ in-crossed single cell zebrafish embryos. Each guide was prepared at a final concentration of 1.25 µM and 2 nL were injected into each embryo. Injected embryos were raised to 9 days post fertilization, where they were subsequently fixed and stained for detailed phenotypic analysis. Stained fish were imaged using a Nikon Eclipse 80i compound microscope with a Nikon DS Ri1 camera. Measurements were taken in ImageJ. Transgenic line Tg(*sox10*:kaede)^39^ was also used in this study.

### Whole mount in situ hybridization analyses

The primers used to generate the *rspo3* RNA probe were the forward primer 5′-AACCTGTGGCTTCAAATGG-3′ and reverse primer 5′-TTGTTGTCGCTCATCCAGTA-3′^40^.

The T7 promotor (gaaattaatacgactcactatagg) was added to all reverse primers. The RNA products were confirmed by gel-electrophoresis. WISH in zebrafish was performed as previously described^41^.

### Skeletal staining

Double Alcian blue/Alizarin red S staining on fixed zebrafish was performed as previously described^42^. The sample size (n) is 5 embryos per each group. The zebrafish palate and lower jaw were dissected and mounted in 4% methyl cellulose prior to imaging. Tartrate- resistant acid phosphatase (TRAP) staining for osteoclast activity was performed (n = 5 wild-type and 5 *rspo3*^−/−^) as adapted from previous study^43^. Imaging was performed using Nikon Eclipse 80i microscope (Melville, NY, USA) and NIS-Elements Br imaging software version 4.40 (2015). Measurements were taken in ImageJ. In vivo Alizarin red S staining of 9 dpf zebrafish was performed as previously described^44^. Alizarin red S and *sox10:kaede* fluorescence was imaged using a Leica SP8 inverted confocal laser scanning microscope. Maximum intensity projections of z-stacks were generated using ImageJ version 2.0.

### RNAscope in situ hybridization, immunofluorescence and confocal imaging

For sample preparation, 48 hpf and 5 dpf zebrafish embryos were fixed using 4% formaldehyde overnight (ON) at 4 °C. Adult zebrafish (6 months old) were fixed using 4% formaldehyde ON at 4 °C and then decalcified ON using 0.35 M EDTA as previously published^45^. The E13.5 and E15.5 mouse embryos were fixed with 4% formaldehyde ON. n = 3 zebrafish embryos and n = 3 mouse embryos were analyzed.

Subsequently, all samples were placed in 15% sucrose in PBS until the tissue sank, and then placed in 30% sucrose in PBS ON. Samples were then embedded in OCT (Tissue-Tek) and serially sectioned (10 um) in coronal orientation using a Leica CM1850 cryostat.

RNAscope probes included: Dr-rspo3-C2 (catalog number: 555121-C2), Dr-runx2a-C1 (catalog number: 409521), Dr-rspo2-C3 (catalog number: 899271-C3) Mm-Rspo3-C3 (catalog number: 402011-C3), Mm-rspo2-C2 (catalog number: 402008-C2). All probes were manufactured by Advanced Cell Diagnostics in Newark, NJ, USA. Sample pre-treatment and RNAscope were performed according to the manufacturer’s instructions (Advanced Cell Diagnostics, Newark, NJ, USA). Stained slides were imaged using a Leica SP8 inverted confocal laser scanning microscope and image processing was performed using ImageJ version 2.0 (2018). Immunofluorescence detection of mouse Runx2 (Abcam primary antibody, catalog number: ab192256; Invitrogen Alexa Fluor 488 goat anti rabbit secondary antibody) was performed following RNAscope in situ hybridization as described by Advanced Cell Diagnostics.

### Micro-computed tomography

Wild-type and *rspo3* mutant adult zebrafish were sacrificed at 6 months of age, n = 9 wild-type and 7 mutant zebrafish. All zebrafish were scanned as previously described^46^. The voxel size of Micro-CT analysis is 10.5 μm. The examiner (K.W.) was blinded to the genotype of the zebrafish. Images were reconstructed, analyzed and viewed using Amira software version 6.

### Measurement of bone volume

The reconstructed bitmap image (BMP) files were converted to NIfTI format for simplification, using Amira software. The threshold tool values were consistent between the samples (32–72 threshold logic unit). Each zebrafish skull was segmented into bone elements (dentary, anguloarticular, premaxilla, maxilla and parasphenoid) using Amira manufacture’s instruction. n = 9 wild type and 7 mutant zebrafish were analyzed at 6 months of age.

### Quantitative RT-PCR

Three independent samples of wild-type and *rspo3* CRISPR/Cas9 (− 20 base pairs micro-deletion mutants) at 6 hpf were collected and measured in triplicate in order to characterize the *rspo3* mutant. We decided to collect embryos at 6 hpf, because it has been reported that *rspo3* mRNA is highly expressed in zebrafish embryos at this time point^34^. In addition, three independent 1-cell stage and 24 hpf wild type embryo samples were collected and measured to define the expression of *rspo3* mRNAs. RNA extractions were performed using RNeasy Mini Kit (Qiagen). SuperScript First-Strand Synthesis System IV (Thermo Fisher Scientific) was used to synthesize first-strand cDNA. Quantitative reverse-transcription PCR (qRT-PCR) was performed using *rspo3* Taqman assay (Dr03109282_m1) Taqman Fast Advanced master mix (Thermo Fisher Scientific) and normalized to 18S rRNA expression (Hs03003631_g1). qPCR was performed on a StepOnePlus Real-Time PCR system (Applied Biosystems).

### Statistical analysis

IBM SPSS statistics version 26 was used for all Student’s t-test statistical analyses. Student’s t-test was used to compare between the two groups. Prism 9 software was used to perform Kruskal–Wallis statistical test with multiple comparisons when more than two groups were compared. Statistical significance was set at p-value ≤ 0.05. Asterisks in the figures indicate p-value ≤ 0.05. Data presented as means ± SEM.

## Supplementary Information

Supplementary Video Legends.

Supplementary Figures.

Supplementary Video 1.

Supplementary Video 2.

## References

[1] Baron R, Kneissel M (2013). WNT signaling in bone homeostasis and disease: From human mutations to treatments. Nat Med..

[2] Boyden LM, Mao J, Belsky J (2002). High bone density due to a mutation in LDL-receptor-related protein 5. N. Engl. J. Med..

[3] Little RD, Carulli JP, Del Mastro RG (2002). A mutation in the LDL receptor-related protein 5 gene results in the autosomal dominant high-bone-mass trait. Am. J. Hum. Genet..

[4] Glass DA, Karsenty G (2006). Molecular bases of the regulation of bone remodeling by the canonical Wnt signaling pathway. Curr. Top. Dev. Biol..

[5] Albers J, Keller J, Baranowsky A (2013). Canonical Wnt signaling inhibits osteoclastogenesis independent of osteoprotegerin. J. Cell Biol..

[6] Vendrell V, Summerhurst K, Sharpe J, Davidson D, Murphy P (2009). Gene expression analysis of canonical Wnt pathway transcriptional regulators during early morphogenesis of the facial region in the mouse embryo. Gene Exp. Patterns..

[7] Brugmann SA, Goodnough LH, Gregorieff A (2007). Wnt signaling mediates regional specification in the vertebrate face. Development.

[8] Geetha-Loganathan P, Nimmagadda S, Antoni L (2009). Expression of WNT signalling pathway genes during chicken craniofacial development. Dev. Dyn..

[9] Alexander C, Piloto S, Le Pabic P, Schilling TF (2014). Wnt signaling interacts with bmp and edn1 to regulate dorsal-ventral patterning and growth of the craniofacial skeleton. PLoS Genet..

[10] Nakajima M, Kou I, Ohashi H, Ikegawa S (2016). Identification and functional characterizationof RSPO2 as a susceptibility gene for ossification of the posterior longitudinal ligament of the spine. Am. J. Hum. Genet..

[11] Estrada K, Styrkarsdottir U, Evangelou E (2012). Genome-wide meta-analysis identifies 56 bone mineral density loci and reveals 14 loci associated with risk of fracture. Nat. Genet..

[12] Kim KA, Zhao J, Andarmani S (2006). R-Spondin proteins: A novel link to beta-catenin activation. Cell Cycle.

[13] Cruciat CM, Niehrs C (2013). Secreted and transmembrane wnt inhibitors and activators. Cold Spring Harb. Perspect. Biol..

[14] Hao HX, Xie Y, Zhang Y (2012). ZNRF3 promotes Wnt receptor turnover in an R-spondin-sensitive manner. Nature.

[15] Lebensohn AM, Rohatgi R (2018). R-spondins can potentiate WNT signaling withoutLGRs. Elife..

[16] Knight MN, Hankenson KD (2014). R-spondins: Novel matricellular regulators of the skeleton. Matrix Biol..

[17] Aoki M, Mieda M, Ikeda T, Hamada Y, Nakamura H, Okamoto H (2007). R-spondin3 is required for mouse placental development. Dev. Biol..

[18] Correa-Rodriguez M, Schmidt Rio-Valle J, Rueda-Medina B (2018). The RSPO3 gene as genetic markers for bone mass assessed by quantitative ultrasound in a population of young adults. Ann. Hum. Genet..

[19] Vieira AR, McHenry TG, Daack-Hirsch S, Murray JC, Marazita ML (2008). Candidate gene/loci studies in cleft lip/palate and dental anomalies finds novel susceptibility genes for clefts. Genet. Med..

[20] Neufeld S, Rosin JM, Ambasta A (2012). A conditional allele of Rspo3 reveals redundant function of R-spondins during mouse limb development. Genesis.

[21] Jin YR, Yoon JK (2012). The R-spondin family of proteins: Emerging regulators of WNT signaling. Int. J. Biochem. Cell. Biol..

[22] Liu F, Millar SE (2010). Wnt/beta-catenin signaling in oral tissue development and disease. J. Dent. Res..

[23] Huysseune A, Soenens M, Elderweirdt F (2014). Wnt signaling during tooth replacement in zebrafish (*Danio rerio*): Pitfalls and perspectives. Front. Physiol..

[24] Thesleff I (2003). Epithelial-mesenchymal signalling regulating tooth morphogenesis. J. Cell Sci..

[25] Liu F, Chu EY, Watt B (2008). Wnt/beta-catenin signaling directs multiple stages of tooth morphogenesis. Dev. Biol..

[26] Kuang-Hsien HuJ, Mushegyan V, Klein OD (2014). On the cutting edge of organ renewal: Identification, regulation, and evolution of incisor stem cells. Genesis.

[27] Tucker A, Sharpe P (2004). The cutting-edge of mammalian development; how the embryo makes teeth. Nat. Rev. Genet..

[28] Yelick PC, Schilling TF (2002). Molecular dissection of craniofacial development using zebrafish. Crit. Rev. Oral. Biol. Med..

[29] Hoshijima K, Jurynec MJ, Klatt Shaw D, Jacobi AM, Behlke MA, Grunwald DJ (2019). Highly efficient CRISPR-Cas9-based methods for generating deletion mutations and F0 embryos that lack gene function in zebrafish. Dev. Cell..

[30] Baniwal SK, Shah PK, Shi Y (2012). Runx2 promotes both osteoblastogenesis and novel osteoclastogenic signals in ST2 mesenchymal progenitor cells. Osteoporos Int..

[31] Zhang M, Zhang P, Liu Y (2017). RSPO3-LGR4 regulates osteogenic differentiation of human adipose-derived stem cells via ERK/FGF signalling. Sci. Rep..

[32] Cobourne MT, Sharpe PT (2010). Making up the numbers: The molecular control of mammalian dental formula. Semin. Cell. Dev. Biol..

[33] Tummers M, Thesleff I (2003). Root or crown: A developmental choice orchestrated by the differential regulation of the epithelial stem cell niche in the tooth of two rodent species. Development.

[34] Verstraeten B, van Hengel J, Huysseune A (2016). Beta-catenin and plakoglobin expression during zebrafish tooth development and replacement. PLoS ONE.

[35] da Fontoura CS, Miller SF, Wehby GL (2015). Candidate gene analyses of skeletal variation in malocclusion. J. Dent. Res..

[36] Pallos D, Hart PS, Cortelli JR (2001). Novel COL1A1 mutation (G559C) [correction of G599C] associated with mild osteogenesis imperfecta and dentinogenesis imperfecta. Arch. Oral. Biol..

[37] Gistelinck C, Kwon RY, Malfait F (2018). Zebrafish type I collagen mutants faithfully recapitulate human type I collagenopathies. Proc. Natl. Acad. Sci. USA.

[38] Kimmel CB, Ballard WW, Kimmel SR, Ullmann B, Schilling TF (1995). Stages of embryonic development of the zebrafish. Dev. Dyn..

[39] Dougherty M, Kamel G, Grimaldi M (2013). Distinct requirements for wnt9a and irf6 in extension and integration mechanisms during zebrafish palate morphogenesis. Development.

[40] Rong X, Chen C, Zhou P (2014). R-spondin 3 regulates dorsoventral and anteroposterior patterning by antagonizing Wnt/beta-catenin signaling in zebrafish embryos. PLoS ONE.

[41] Ling IT, Rochard L, Liao EC (2017). Distinct requirements of wls, wnt9a, wnt5b and gpc4 in regulating chondrocyte maturation and timing of endochondral ossification. Dev. Biol..

[42] Walker MB, Kimmel CB (2007). A two-color acid-free cartilage and bone stain for zebrafish larvae. Biotech. Histochem..

[43] Hammond CL, Schulte-Merker S (2009). Two populations of endochondral osteoblasts with differential sensitivity to Hedgehog signalling. Development.

[44] Bensimon-Brito A, Cardeira J, Dionisio G, Huysseune A, Cancela ML, Witten PE (2016). Revisiting in vivo staining with alizarin red S: a valuable approach to analyse zebrafish skeletal mineralization during development and regeneration. BMC Dev. Biol..

[45] Copper JE, Budgeon LR, Foutz CA (2018). Comparative analysis of fixation and embedding techniques for optimized histological preparation of zebrafish. Comp. Biochem. Physiol. C..

[46] Charles JF, Sury M, Tsang K (2017). Utility of quantitative micro-computed tomographic analysis in zebrafish to define gene function during skeletogenesis. Bone.

