# Supplementary material for: Synergistic roles of Wnt modulators R-spondin2 and R-spondin3 in craniofacial morphogenesis and dental development
Source: Sci Rep. 2021 Mar 12;11:5871. doi: 10.1038/s41598-021-85415-y (PMC7954795; doi:10.1038/s41598-021-85415-y)
Supplement: Supplementary file 1 — Supplementary Video Legends. [file 41598_2021_85415_MOESM1_ESM.docx]

Supplementary Videos

**Synergistic roles of Wnt Modulators *R-spondin2 and R-spondin3* in Craniofacial Morphogenesis and Dental Development**

Nora Alhazmi^1,†^, Shannon H. Carroll^2,3,4†^, Kenta Kawasaki^2,3^, Katherine C. Woronowicz^5,6^, Shawn A. Hallett^2,4^, Claudio Macias Trevino^2,4^, Edward B. Li^2,4^, Roland Baron^1,4^, Francesca Gori^1^, Pamela C. Yelick^7^, Matthew P. Harris^5,6^, and Eric C. Liao^2,3,4,8*^

^1^ Harvard School of Dental Medicine, Boston, Massachusetts, United States of America.

^2^ Center for Regenerative Medicine, Massachusetts General Hospital, Boston, Massachusetts, United States of America.

^3^ Shriners Hospital for Children, Boston, Massachusetts, United States of America.

^4^ Department of Medicine, Harvard Medical School, Boston, Massachusetts, United States of America.

^5^ Department of Genetics, Harvard Medical School, Boston, Massachusetts, United States of America.

^6^ Department of Orthopedics, Boston Children’s Hospital, Boston, Massachusetts, United States of America.

^7^ Department of Orthodontics, Division of Craniofacial and Molecular Genetics, Tufts University School of Dental Medicine, Boston, Massachusetts, United States of America.

^8^ Division of Plastic and Reconstructive Surgery, Massachusetts General Hospital, Boston, Massachusetts, United States of America.

^†^ These authors contributed equally to this work.

**Supplemental Video S1. Micro-CT of wild-type zebrafish with color coded bone elements.**

Micro-CT image of wild type zebrafish with color coded bone elements (blue: parashenoid, pink: maxilla, yellow: premaxilla, green: dentary and red: anguloarticular).

**Supplemental Video S2. Micro-CT of *rspo3*-/- zebrafish with color coded bone elements.**

Micro-CT image of *rspo3* mutant zebrafish showing midface deficiency with color coded bone elements (blue: parashenoid, pink: maxilla, yellow: premaxilla, green: dentary and red: anguloarticular).
