# Supplementary material for: Synergistic roles of Wnt modulators R-spondin2 and R-spondin3 in craniofacial morphogenesis and dental development
Source: Sci Rep. 2021 Mar 12;11:5871. doi: 10.1038/s41598-021-85415-y (PMC7954795; doi:10.1038/s41598-021-85415-y)
Supplement: Supplementary file 2 — Supplementary Figures. [file 41598_2021_85415_MOESM2_ESM.docx]

Supplementary Figure

**Synergistic roles of Wnt Modulators *R-spondin2 and R-spondin3* in Craniofacial Morphogenesis and Dental Development**

Nora Alhazmi^1,†^, Shannon H. Carroll^2,3,4†^, Kenta Kawasaki^2,3^, Katherine C. Woronowicz^5,6^, Shawn A. Hallett^2,4^, Claudio Macias Trevino^2,4^, Edward B. Li^2,4^, Roland Baron^1,4^, Francesca Gori^1^, Pamela C. Yelick^7^, Matthew P. Harris^5,6^, and Eric C. Liao^2,3,4,8*^

^1^ Harvard School of Dental Medicine, Boston, Massachusetts, United States of America.

^2^ Center for Regenerative Medicine, Massachusetts General Hospital, Boston, Massachusetts, United States of America.

^3^ Shriners Hospital for Children, Boston, Massachusetts, United States of America.

^4^ Department of Medicine, Harvard Medical School, Boston, Massachusetts, United States of America.

^5^ Department of Genetics, Harvard Medical School, Boston, Massachusetts, United States of America.

^6^ Department of Orthopedics, Boston Children’s Hospital, Boston, Massachusetts, United States of America.

^7^ Department of Orthodontics, Division of Craniofacial and Molecular Genetics, Tufts University School of Dental Medicine, Boston, Massachusetts, United States of America.

^8^ Division of Plastic and Reconstructive Surgery, Massachusetts General Hospital, Boston, Massachusetts, United States of America.

^†^ These authors contributed equally to this work.


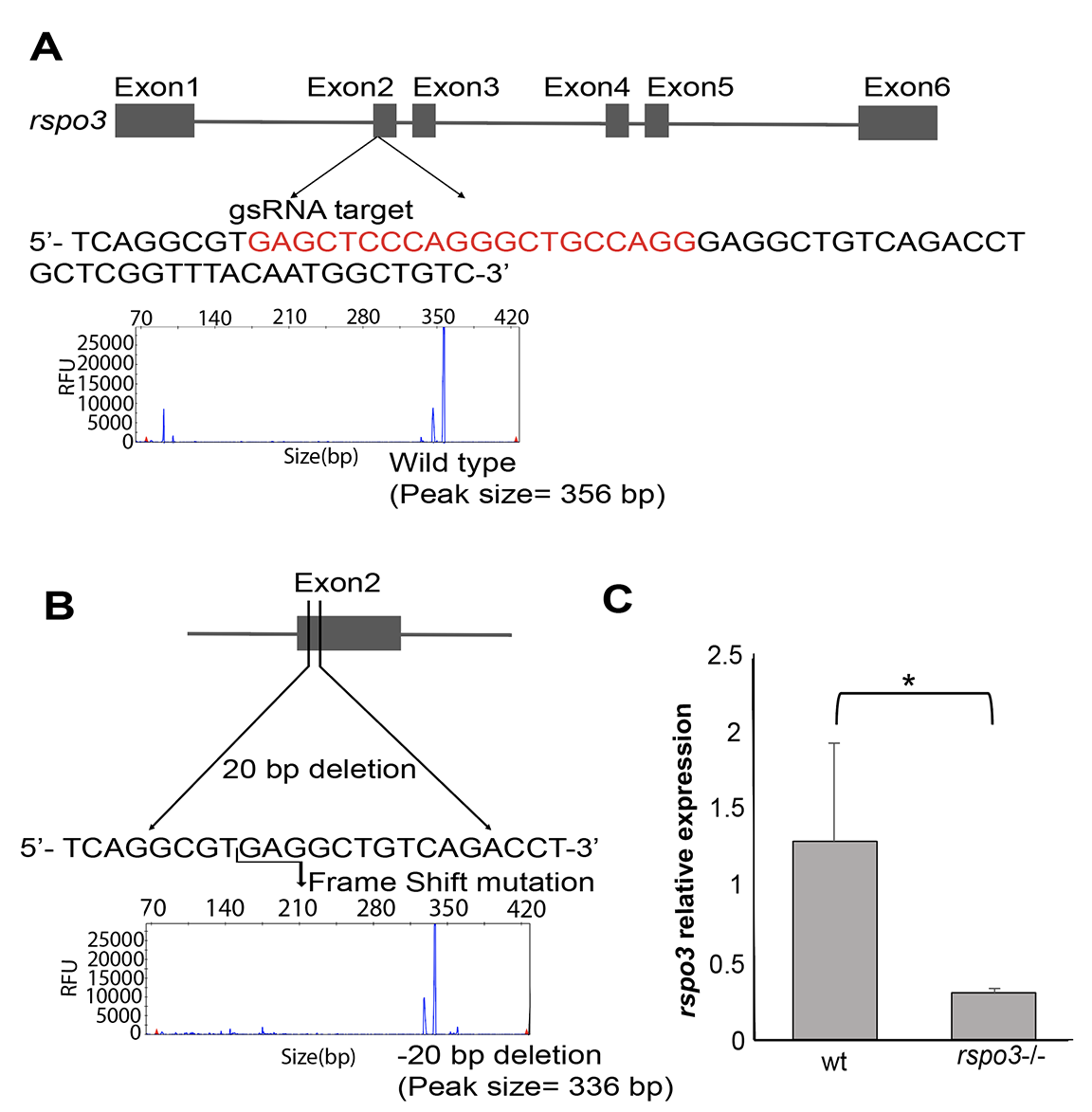


**Supplemental Figure S1. Generation and characterization of CRISPR-Cas targeted *rspo3* allele in zebrafish.** A) Guided RNA target is designed in *rspo3* exon 2 at the indicated sequence (red) for CRISPR-Cas mediated gene editing. Microsatellite genotyping presenting the wild-type allele with peak size=356 bp. B) Frame shift mutation is generated with -20 base pair deletion.

Microsatellite genotyping presenting the mutant allele with peak size = 336 bp. C) Characterization of *rspo3*-/- and *rspo3*+/- by RT-qPCR at 6 hpf. The mRNA levels are significantly reduced in *rspo3*-/-, likely consequence of mRNA nonsense mediated decay. *p ≤

0.05. *rspo3* mRNA is reduced in *rspo3*-/- compared to wild-type. However, rspo3 mRNA levels are not statistically significant between *rspo3*+/- and wild-type. RFU: Relative fluorescence units.
